# Current Awareness on Comparative and Functional Genomics

**DOI:** 10.1002/cfg.116

**Published:** 2002-02


**Comparative and Functional Genomics**
*Comp. Funct. Genom.* 2002; **3:** 81–88
**DOI**:10.1002/cfg.116

### Current awareness on comparative and functional genomics

### 1 Reviews & symposia

Ashton PD, Curwen RS, Wilson RA. 2001. Univ York, Dept Biol, POB 373, York YO10 5YW, England. Linking proteome and genome: How to identify parasite proteins (Review). *Trends Parasitol* **17:** (4) 198.

Bakhtiar R, Nelson RW. 2001. Merck Res Labs, Dept Drug Metab, Mail Stop, Rahway, NJ 07065, USA. Mass spectrometry of the proteome (Minireview). *Mol Pharmacol* **60:** (3) 405.

Bumann D, Meyer TF, Jungblut PR. 2001. Max Planck Inst Infect Biol, Dept Mol Biol, Schumannstr 21-22, DE-10117 Berlin, Germany. Proteome analysis of the common human pathogen *Helicobacter pylori* (Review). *Proteomics* **1:** (4) 473.

Cavalcoli JD. 2001. Pfizer Global Res & Dev, Ann Arbor Labs, 2800 Plymouth Rd, Ann Arbor, Mi 48105, USA. Genomic and proteomic databases: Large-scale analysis and integration of data (Review). *Trends Cardiovasc Med* **11:** (2) 76.

Cordwell SJ, Nouwens AS, Walsh BJ. 2001. MacQuarie Univ, Australian Proteome Anal Facil, Level 4, Bldg F7B, Sydney, NSW 2109, Australia. Comparative proteomics of bacterial pathogens (Review). *Proteomics* **1:** (4) 461.

Cronk QCB. 2001. Univ Edinburgh, Inst Cell & Mol Biol, Kings Bldg, Mayfield Rd, Edinburgh EH9 3JH, Scotland. Plant evolution and development in a post-genomic context (Review). *Nat Rev Genet* **2:** (8) 607.

Dean PM, Zanders ED, Bailey DS. 2001. De Novo Pharmaceut Ltd, St Andrews House, 59 St Andrews St, Cambridge CB2 3DD, England. Industrial-scale, genomics-based drug design and discovery (Opinion). *Trends Biotechnol* **19:** (8) 288.

Dent RM, Han M, Niyogi KK. 2001. Univ Calif, Dept Plant & Microbial Biol, Berkeley, Ca 94720, USA. Functional genomics of plant photosynthesis in the fast lane using *Chlamydomonas reinhardtii* (Review). *Trends Plant Sci* **6:** (8) 364.

Dwek MV, Ross HA, Leathem AJC. 2001. Royal Free & UCL, Med Sch, Inst Surg Studies, Dept Surg, Charles Bell House, London W1W 1EJ, England. Proteome and glycosylation mapping identifies post-translational modifications associated with aggressive breast cancer: Review. *Proteomics* **1:** (6) 756.

Green ED. 2001. NIH/NHGRI, Genome Technol Branch, Bethesda, Md 20892, USA. Strategies for the systematic sequencing of complex genomes (Review). *Nat Rev Genet* **2:** (8) 573.

Guengerich FP. 2001. Vanderbilt Univ, Sch Med, Dept Biochem, Nashville, Tn 37232, USA. Functional genomics and proteomics applied to the study of nutritional metabolism. *Nutr Rev* **59:** (8) 259.

Hashmi S, Tawe W, Lustigman S*. 2001. *New York Blood Ctr, Lindsley F Kimball Res Inst, Mol Parasitol Lab, 310 East 67th St, New York, NY 10021, USA. *Caenorhabditis elegans* and the study of gene function in parasites (Review). *Trends Parasitol* **17:** (8) 387.

Haslam SM, Morris HR, Dell A*. 2001. *Univ London Imperial Coll Sci Technol & Med, Dept Biochem, London SW7 2AY, England. Mass spectrometric strategies: Providing structural clues for helminth glycoproteins (Review). *Trends Parasitol* **17:** (5) 231.

Hooper NM. 2001. Univ Leeds, Sch Biochem & Mol Biol, Leeds LS2 9JT, England. Determination of glycosyl-phosphatidylinositol membrane protein anchorage: Review. *Proteomics* **1:** (6) 748.

Heinemann U, Illing G, Oschkinat H. 2001. Max-Delbruck-Ctr Mol Med, Forsch Grp Kristallog, Robert Rossle Str 10, DE-13125 Berlin, Germany. High-throughput three-dimensional protein structure determination. *Curr Opin Biotechnol* **12:** (4) 348.

Jeon JS, An GH*. 2001. *Pohang Univ Sci & Technol, Div Mol & Life Sci, Natl Res Lab Plant Funct Genomics, Pohang 790784, South Korea. Gene tagging in rice: A high throughput system for functional genomics (Review). *Plant Sci* **161:** (2) 211.

Jonsson AP. 2001. Karolinska Inst, Dept Med Biochem & Biophys, SE-17177 Stockholm, Sweden. Mass spectrometry for protein and peptide characterisation (Review). *Cell Mol Life Sci* **58:** (7) 868.

Kell DB, Darby RM, Draper J. 2001. Univ Wales, Inst Biol Sci, Aberystwyth SY23 3DD, Wales. Genomic computing. Explanatory analysis of plant expression profiling data using machine learning. *Plant Physiol* **126:** (3) 943.

Labigne A. 2001. Inst Pasteur, Unite Pathogenie Bacterienne Muqueuses, 28 rue Dr Roux, FR-75724 Paris 15, France. The *Helicobacter pylori* genomes: New insights into physiopathology and therapeutic (Review). *M S-Med Sci* **17:** (6-7) 712.

Larocca D, Baird A. 2001. Selective Genetics, 11035 Roselle St, San Diego, Ca 92121, USA. Receptor-mediated gene transfer by phage-display vectors: Applications in functional genomics and gene therapy (Review). *Drug Discov Today* **6:** (15) 793.

Lecompte O, Thompson JD, Plewniak F, Thierry JC, Poch O*. 2001. *ULP, INSERM, CNRS, Inst Genet & Biol Mol Cellulaire, Lab Biol & Genet Struct, BP 163, FR-67404 Illkirch Graffenstaden, France. Multiple alignment of complete sequences (MACS) in the post-genomic era (Review). *Gene* **270:** (1-2) 17.

Lesley SA. 2001. Novartis Res Fdn, Genomics Inst, 3115 Merryfield Row, San Diego, Ca 92121, USA. High-throughput proteomics: Protein expression and purification in the postgenomic world. *Protein Expr Purif* **22:** (2) 159.

Macri J, Rapundalo ST*. 2001. *Pfizer Global Res & Dev, Dept Cardiovasc Mol Sci, 2800 Plymouth Rd, Ann Arbor, Mi 48105, USA. Application of proteomics to the study of cardiovascular biology (Review). *Trends Cardiovasc Med* **11:** (2) 66.

Mann M, Hendrickson RC, Pandey A. 2001. Univ Sthn Denmark, Dept Biochem & Mol Biol, Protein Interact Lab, Campusvej 55, DK-5230 Odense M, Denmark. Analysis of proteins and proteomes by mass spectrometry. *Annu Rev Biochem* **70:** 437.

Marcotte ER, Srivastava LK, Quirion R. 2001. McGill Univ, Douglas Hosp, Fac Med, Dept Psychiat, Res Ctr, Verdun, Quebec, Canada H4H 1R3. DNA microarrays in neuropsychopharmacology (Review). *Trends Pharmacol Sci* **22:** (8) 426.

Mirnics K, Middleton FA, Lewis DA, Levitt P. 2001. Univ Pittsburgh, Sch Med, Dept Psychiatry, Pittsburgh, Pa 15261, USA. Analysis of complex brain disorders with gene expression microarrays: Schizophrenia as a disease of the synapse (Review). *Trends Neurosci* **24:** (8) 479.

Mittl PRE, Grutter MG. 2001. Univ Zurich, Inst Biochem, Winterthurerstr 190, CH-8057 Zurich, Switzerland. Structural genomics: Opportunities and challenges. *Curr Opin Chem Biol* **5:** (4) 402.

Naaby-Hansen S, Waterfield MD, Cramer R. 2001. Royal Free & UCL Med Sch, Ludwig Inst Canc Res, 91 Riding House St, London W12 7BS, England. Proteomics: Post-genomic cartography to understand gene function (Review). *Trends Pharmacol Sci* **22:** (7) 376.

Nalivaeva NN, Turner AJ*. 2001. *Univ Leeds, Sch Biochem & Mol Biol, Leeds LS2 9JT, England. Post-transitional modifications of proteins: Acetylcholinesterase as a model system (Review). *Proteomics* **1:**

In order to keep subscribers up-to-date with the latest developments in their field, this current awareness service is provided by John Wiley & Sons and contains newly-published material on comparative and functional genomics. Each bibliography is divided into 16 sections. 1 Reviews & symposia; 2 General; 3 Large-scale sequencing and mapping; 4 Evolutionary genomics; 5 Comparative genomics; 6 Pathways, gene families and regulons; 7 Pharmacogenomics; 8 EST, cDNA and other clone resources; 9 Functional genomics; 10 Transcriptomics; 11 Proteomics; 12 Protein structural genomics; 13 Metabolomics; 14 Genomic approaches to development; 15 Technological advances; 16 Bioinformatics. Within each section, articles are listed in alphabetical order with respect to author. If, in the preceding period, no publications are located relevant to any one of these headings, that section will be omitted.

Copyright © 2002 John Wiley & Sons, Ltd.

(6) 735.

Pelletier J, Sidhu S. 2001. Univ Montreal, Dept Chim, 2900 Edouard Montpetit, Montreal, Quebec, Canada H3C 3J7. Mapping protein-protein interactions with combinatorial biology methods. *Curr Opin Biotechnol* **12:** (4) 340.

Peng J, Gygi SP*. 2001. *Harvard Med Sch, Dept Cell Biol, 240 Longwood Ave, Boston, Ma 02115, USA. Special feature: Tutorial - Proteomics: The move to mixtures. *J Mass Spectrom* **36:** (10) 1083.

Prestegard JH, Valafar H, Glushka J, Tian F. 2001. Univ Georgia, Complex Carbohydrate Res Ctr, Athens, Ga 30602, USA. Nuclear magnetic resonance in the era of structural genomics. *Biochemistry* **40:** (30) 8677.

Sawyer TK. 2001. ARIAD Pharmaceut Inc, Cambridge, Ma 02139, USA. Proteomics: Structure and function. *Biotechniques* **31:** (1) 156.

Stanton LW. 2001. Geron Corp, 230 Constitution Dr, Menlo Park, Ca 94025, USA. Methods to profile gene expression (Review). *Trends Cardiovasc Med* **11:** (2) 49.

Sweigard JA, Ebbole DJ. 2001. Delaware Technol Pk, Suite 200, 1 Innovation Way, POB 6, Newark, De 19714, USA. Functional analysis of pathogenicity genes in a genomics world. *Curr Opin Microbiol* **4:** (4) 387.

Tasara T, Hottiger MO, Hubscher U*. 2001. *Univ Zurich Inst Vet Biochem, Winterthurerstr 190, CH-8057 Zurich, Switzerland. Functional genomics in HIV-1 virus replication: Protein-protein interactions as a basis for recruiting the host cell machinery for viral propagation (Review). *Biol Chem* **382:** (7) 993.

Vercoutter-Edouart AS, Peyrat JP*, Lemoine J, Hondermarck H. 2001. *Univ Sci & Technol Lille, Lab Chim Biol, UMR 111, FR-59655 Villeneuve d'Ascq, France. Proteomic analysis: Why and how? (Review) (French, English Abstract). *Bull Cancer* **88:** (7) 663.

White KP. 2001. Yale Univ, Sch Med, Dept Genet, New Haven, Ct 06510, USA. Functional genomics and the study of development, variation and evolution (Review). *Nat Rev Genet* **2:** (7) 528.

Wing BA, Browne EP, Shenk T. 2001. Princeton Univ, Dept Mol Biol, Princeton, NJ 08544, USA. DNA microarrays: A powerful new tool for analysis of the virus-host interaction (Review). *Drug Discov Today* **6:** (15 Suppl) S67.

Xenarios L, Eisenberg D. 2001. UCLA, DOE Lab Struct Biol & Mol Med, POB 951570, Los Angeles, Ca 90095, USA. Protein interaction databases. *Curr Opin Biotechnol* **12:** (4) 334.

### 2 General

Bond U, Campbell SG, James TC. 2001. Univ Dublin, Trinity Coll, Dept Microbiol, Moyne Inst Prevent Med, Dublin 2, Rep Ireland. A model organism of genomic and postgenomic studies. *IEEE Eng Med Biol Mag* **20:** (4) 22.

Brower V. 2001. Address not available. Proteomics: Biology in the post-genomic era - Companies all over the world rush to lead the way in the new post-genomics race. *EMBO Rep* **2:** (7) 558.

Greenbaum D, Luscombe NM, Jansen R, Qian J, Gerstein M*. 2001. *Yale Univ, Dept Mol Biophys & Biochem, New Haven, Ct 06520, USA. Interrelating different types of genomic data, from proteome to secretome: 'Oming in on function. *Genome Res* **11:** (9) 1463.

### 3 Large-scale sequencing and mapping

Galibert F, Finan TM, Long SR*, Puhler A, Abola P, Ampe F, Barloy-Hubler F, Barnett MJ, Becker A, Boistard P, et al. 2001. *Stanford Univ, Dept Biol Sci, Stanford, Ca 94305, USA. The composite genome of the legume symbiont *Sinorhizobium meliloti*. *Science* **293:** (5530) 668.

Ke XY, Tapper W, Collins A. 2001. Univ Southampton, Gen Hosp, Human Genet Res Div, Duthie Bldg, Mailpoint 808, Tremona Rd, Southampton SO16 6YD, England. LDB2000: Sequence-based integrated maps of the human genome. *Bioinformatics* **17:** (7) 581.

Lim A, Dimalanta ET, Potamousis KD, Yen G, Apodoca J, Tao CH, Lin JY, Qi R, Skiadis J, Ramanathan A, Perna NT, Plunkett G, Burland V, Mau B, Hackett J, Blattner FR, Anantharaman TS, Mishra B, Schwartz DC*. 2001. *Univ Wisconsin, Lab Mol & Computat Genomics, Madison, Wi 53706, USA. Shotgun optical maps of the whole *Escherichia coli* O157:H7 genome. *Genome Res* **11:** (9) 1584.

Oudott-le-Secq MP, Fontaine JM, Rousvoal S, Kloareg B, Loiseaux de Goer S*. 2001. *CNRS UMR 1931, BP 74, FR-29682 Roscoff, France. The complete sequence of a brown algal mitochondrial genome, the ectocarpale *Phylaiella littoralis* (L.) Kjelm. *J Mol Evol* **53:** (2) 80.

Rouillard JM, Erson AE, Kuick R, Asakawa J, Wimmer K, Muleris M, Petty EM, Hanash S*. 2001. *Univ Michigan, Dept Pediat, Ann Arbor, Mi 48109, USA. Virtual genome scan: A tool for restriction landmark-based scanning of the human genome. *Genome Res* **11:** (8) 1453.

Skovgaard M, Jensen LJ, Brunak S, Ussery D, Krogh A. 2001. Tech Univ Denmark, Bioctr, Ctr Biol Sequence Anal, Bldg 208, DK-2800 Lyngby, Denmark. On the total number of genes and their length distribution in complete microbial genomes. *Trends Genet* **17:** (8) 425.

Tomkins JP, Wood TC, Stacey MG, Loh JT, Judd A, Goicoechea JL, Stacey G, Sadowsky MJ, Wing RA. 2001. Clemson Univ, Genomics Inst, Clemson, SC 29634, USA. A marker-dense physical map of the *Bradyrhizobium japonicum* genome. *Genome Res* **11:** (8) 1434.

Wendl MC, Korf I, Chinwalla AT, Hillier LW. 2001. Washington Univ, Gen Sequencing Ctr, 4444 Forest Pk Blvd, Box 8501, St Louis, Mo 63108, USA. Automated processing of raw DNA sequence data. *IEEE Eng Med Biol Mag* **20:** (4) 41.

### 4 Evolutionary genomics

Egan S, Wiener P, Kallifidas D, Wellington EMH*. 2001. *Univ Warwick, Dept Biol Sci, Coventry CV4 7AL, England. Phylogency of *Streptomyces* species and evidence for horizontal transfer of entire and partial antibiotic gene clusters. *Antonie van Leeuwenhoek* **79:** (2) 127.

Fitzgerald JR, Sturdevant DE, Mackie SM, Gill SR, Musser JM*. 2001. *NIH/NIAID, Rocky Mt Labs, Lab Human Bacterial Pathogenesis, 903 Sth 4th St, Hamilton, Mt 59840, USA. Evolutionary genomics of *Staphylococcus aureus*: Insights into the origin of methicillin-resistant strains and the toxic shock syndrome epidemic. *Proc Natl Acad Sci U S A* **98:** (15) 8821.

Herniou EA, Luque T, Chen X, Vlak JM, Winstanley D, Cory JS, O'Reilly D*. 2001. *Univ London, Imperial Coll Sci Technol & Med, Dept Biol, Imperial Coll Rd, London SW7 2AZ, England. Use of whole genome sequence data to infer baculovirus phylogeny. *J Virol* **75:** (17) 8117.

Katti MV, Ranjekar PK, Gupta VS*. 2001. *Natl Chem Lab, Div Biochem Sci, Plant Mol Biol Unit, IN-411008 Pune, India. Differential distribution of simple sequence repeats in eukaryotic genome sequences. *Mol Biol Evol* **18:** (7) 1161.

Makarenkov U. 2001. Univ Montreal, Dept Sci Biol, CP 6128, Succ Ctr-Ville, Montreal, Quebec, Canada H3C 3J7. T-REX: Reconstructing and visualizing phylogenetic trees and reticulation networks. *Bioinformatics* **17:** (7) 664.

Suzuki Y, Gojobori T, Nei M. 2001. Penn State Univ, Inst Mol Evolutionary Genet, Mueller Lab 328, University Park, Pa 16802, USA. ADAPTSITE: Detecting natural selection at single amino acid sites. *Bioinformatics* **17:** (7) 660.

Wicker T, Stein N, Albar L, Feuillet C, Schlagenhauf E, Keller B*. 2001. *Univ Zurich, Inst Plant Biol, Zollikerstr 107, CH-8008 Zurich, Switzerland. Analysis of a contiguous 211 kb sequence in diploid wheat (*Triticum monococcum* L.) reveals multiple mechanisms of genome evolution. *Plant J* **26:** (3) 307.

### 5 Comparative genomics

Barloy-Hubler F, Lalaure V, Galibert F*. 2001. *Lab Genet & Dev, UMR 6061 CNRS, 2 Ave Pr Leon Bernard, FR-35043 Rennes, France. Ribosomal protein gene cluster analysis in eubacterium genomics: Homology between *Sinorhizobium meliloti* strain 1021 and *Bacillus subtilis*. *Nucleic Acids Res* **29:** (13) 2747.

Clark MS, Smith SF, Elgar G. 2001. HGMP Resource Ctr, Fugu Genomics, Wellcome Genome Campus, Hinxton, Cambridge CB10 1SB, England. Use of the Japanese pufferfish (*Fugu rubripes*) in comparative genomics. *Mar Biotechnol* **3:** (1) S130.

Copyright © 2002 John Wiley & Sons, Ltd. 

Kondo S, Shinagawa A, Saito T, Kiyosawa H, Yamanaka I, Aizawa K, Fukuda S, Hara A, Itoh M, Kawai J, Shibata K, Hayashizaki Y. 2001. Yokohama Inst, RIKEN Genome Sci Ctr, Lab Genome, Explorat Res Grp, Tsurumi ku, Yokohama, Kanagawa 230 004, Japan. Computational analysis of full-length mouse cDNAs compared with human genome sequences. *Mamm Genome* **12:** (9) 673.

Murray AE, Lies D, Li G, Nealson K, Zhou J, Tiedje JM. 2001. Desert Res Inst, Reno, Nv 89512, USA. DNA/DNA hybridization to microarrays reveals gene-specific differences between closely related microbial genomes. *Proc Natl Acad Sci U S A* **98:** (17) 9853.

Nolling J, Breton G, Omelchenko MV, Makarova KS, Zeng QD, Gibson R, Lee HM, Dubois J, Qiu DY, Hitti J, Wolf YI, Tatusov RL, Sabathe F, Doucette-Stamm L, Soucaille P, Daly MJ, Bennett GN, Koonin EV, Smith DR*. 2001. *Genome Therapeut Corp, GTC Sequencing Ctr, 100 Beaver St, Waltham, Ma 02453, USA. Genome sequence and comparative analysis of the solvent-producing bacterium *Clostridium acetobutylicum*. *J bacteriol* **183:** (16) 4823.

Nonaka M, Matsuo M, Naruse K, Shima A. 2001. Univ Tokyo, Grad Sch Sci, Dept Sci Biol, 7-3-1 Hongo, Tokyo 113 0033, Japan. Comparative genomics of medaka: The major histocompatibility complex (MHC). *Mar Biotechnol* **3:** (1) S141.

Perret X, Parsons J, Viprey V, Reichwald K, Broughton WJ. 2001. Univ Geneva, LBMPS, 1 Chemin Imperatrice, CH-1292 Geneva, Switzerland. Repeated sequences of *Rhizobium* sp NGR234 and *Sinorhizobium meliloti* genomes: A comparative analysis using random sequencing (French, English Abstract). *Can J Microbiol* **47:** (6) 548.

Rivas E, Klein RJ, Jones TA, Eddy SR*. 2001. *Washington Univ, Sch Med, Howard Hughes Med Inst, St Louis, Mo 63110, USA. Computational identification of noncoding RNAs in *E. coli* by comparative genomics. *Curr Biol* **11:** (17) 1369.

Zafar N, Mazumder R, Seto D*. 2001. *George Mason Univ, Sch Computat Sci, 10900 Univ Blvd, Manassas, Va 20110, USA. Comparisons of gene colinearity in genomes using GeneOrder2.0. *Trends Biochem Sci* **26:** (8) 514.

### 6 Pathways, gene families and regulons

Krubasik P, Kobayashi M, Sandmann G*. 2001. *Univ Frankfurt, Inst Bot, Biosynth Grp, POB 111932, DE-60054 Frankfurt, Germany. Expression and functional analysis of a gene cluster involved in the synthesis of decaprenoxanthin reveals the mechanisms for $C_{50}$ carotenoid formation. *Eur J Biochem* **268:** (13) 3702.

McKean PG, Denny PW, Knuepfer E, Keen JK, Smith DF*. 2001. *Univ London, Imperial Coll Sci Technol & Med, Dept Biochem, Wellcome Trust, Labs Mol Parasitol, London SW7 2AY, England. Phenotypic changes associated with deletion and overexpression of a stage-regulated gene family in *Leishmania*. *Cell Microbiol* **3:** (8) 511.

### 7 Pharmacogenomics

Aberger F, Costa-Pereira AP, Schlaak JF, Williams TM, O'Shaughnessy RFL, Hollaus G, Kerr IM, Frischauf AMM*. 2001. *Salzburg Univ, Inst Genet, Hellbrunnerstr 34, AU-5020 Salzburg, Austria. Analysis of gene expression using high-density and IFN-γ-specific low-density cDNA arrays. *Genomics* **77:** (1-2) 50.

Cho YS, Kim MK, Cheadle C, Neary C, Becker KG, Cho-Chung YS*. 2001. *NIH/Cellular Biochem Sect, Tumor Immunol & Biol Lab, Bldg 10, Room 5805, 9000 Rockville Pike, Bethesda, Md 20892, USA. Antisense DNAs as multisite genomic modulators identified by DNA microarray. *Proc Natl Acad Sci U S A* **98:** (17) 9819.

De Vos J, Couderc G, Tarte K, Jourdan M, Requirand G, Delteil MC, Rossi JF, Mechti N, Klein B*. 2001. *INSERM U475, Unit Cellular Therapy, 99 rue Puech Villa, Montpellier 5, France. Identifying intercellular signaling genes expressed in malignant plasma cells by using complementary DNA arrays. *Blood* **98:** (3) 771.

Hampe J, Wollstein A, Lu T, Frevel H J, Will M, Manaster C, Schreiber S. 2001. Univ Kiel, Dept Med 1, DE 24105 Kiel, Germany. An integrated system for high throughput TaqMan® based SNP genotyping. *Bioinformatics* **17:** (7) 654.

Hofmann WK, De Vos S, Tsukasaki K, Wachsman W, Pinkus GS, Said JW, Koeffler HP. 2001. UCLA, Cedars Sinai Med Ctr, Sch Med, Div Hematol & Oncol, 8700 Beverly Bvd, Los Angeles, Ca 90048, USA. Altered apoptosis pathways in mantle cell lymphoma detected by oligonucleotide microarray. *Blood* **98:** (3) 787.

Kihara C, Tsunoda T, Tanaka T, Yamana H, Furukawa Y, Ono K, Kitahara O, Zembutsu H, Yanagawa R, Hirata K, Takagi T, Nakamura Y*. 2001. *Univ Tokyo, Inst Med Sci, Ctr Human Genome, Mol Med Lab, Minato ku, 4-6-1 Shirokanedai, Tokyo 108 8639, Japan. Prediction of sensitivity of esophageal tumors to adjuvant chemotherapy by cDNA microarray analysis of gene-expression profiles. *Cancer Res* **61:** (17) 6474.

Kim JJ. 2001. Viral Henomix Inc, 3600 Market St, Suite 100, Philadelphia, Pa 19104, USA. Using viral genomics to develop viral gene products as a novel class of drugs to treat human ailments. *Biotechnol Lett* **23:** (13) 1015.

Kim JY, Wu H, Hawthorne L, Rafii S, Laurence J. 2001. Cornell Univ, Weill Med Coll, Lab AIDS Virus Res, 515 East 71st St, New York, NY 10021, USA. Endothelial cell apoptotic genes associated with the pathogenesis of thrombotic microangiopathies: An application of oligonucleotide genechip technology. *Microvasc Res* **62:** (2) 83.

Miyazato A, Ueno S, Ohmine K, Ueda M, Yoshida K, Yamashita Y, Kaneko T, Mori M, Kirito K, Toshima M, Nakamura Y, Sato K, Kano Y, Furusawa S, Ozawa K, Mano K*. 2001. *Jichi Med Sch, Div Funct Genomics, 3311-1 Yakushiji, Minami kawachi, Tochigi 329 0498, Japan. Identification of myelodysplastic syndrome-specific genes by DNA microarray analysis with purified hematopoietic stem cell fraction. *Blood* **98:** (2) 422.

Musumarra G, Condorelli DF, Scire S, Costa AS. 2001. Univ Catania, Dipt Sci Chim, Viale A Doria 6, IT-95125 Catania, Italy. Shortcuts in genome-scale cancer pharmacology research from multivariate analysis of the National Cancer Institute gene expression database. *Biochem Pharmacol* **62:** (5) 547.

Okabe S, Fujimoto N, Sueoka N, Suganuma M, Fujiki H*. 2001. *Saitama Canc Ctr, 818 Komuro, INA, Saitama 362 0806, Japan. Modulation of gene expression by (-)-epigallocatechin gallate in PC-9 cells using a cDNA expression array. *Biol Pharm Bull* **24:** (8) 883.

Rannala B, Reeve JP. 2001. Univ Alberta, Dept Med Genet, 8-39 Med Sci Bldg, Edmonton, Alberta, Canada T6G 2H7. High-resolution multipoint linkage-disequilibrium mapping in the context of a human genome sequence. *Am J Hum Genet* **69:** (1) 159.

Ross BC, Czajkowski L, Hocking D, Margetts M, Webb E, Rothel L, Patterson M, Agius C, Camuglia S, Reynolds E, Littlejohn T, Gaeta B, Ng A, Kuczek ES, Mattick JS, Gearing D, Barr IG. 2001. CSL Ltd, Res & Dev, 45 Poplar Rd, Parkville, Vic 3052, Australia. Identification of vaccine candidate antigens from a genomic analysis of *Porphyromonas gingivalis*. *Vaccine* **19:** (30) 4135.

Southan C. 2001. Gemini Genomics UK, 162 Sci Pk, Milton Rd, Cambridge CB4 0GH, England. A genomic perspective on human proteases as drug targets. *Drug Discov Today* **6:** (13) 681.

Tackels-Horne D, Goodman MD, Williams AJ, Wilson DJ, Eskandari T, Vogt LM, Boland JF, Scherf U, Vockley JG*. 2001. *Gene Log Inc, 708 Quince Orchard Rd, Gaithersburg, Md 20878, USA. Identification of differentially expressed genes in hepatocellular carcinoma and metastatic liver tumors by oligonucleotide expression profiling. *Cancer* **92:** (2) 395.

### 8 EST, cDNA and other clone resources

Carninci P, Shibata Y, Hayatsu N, Itoh M, Shiraki T, Hirozane T, Watahiki A, Shibata K, Konno H, Muramatsu M, Hayashizaki Y. 2001. RIKEN Genome Sci Ctr, Genome Explorat Res Grp, Tsurumi ku, 1-7-22 Suehiro cho, Kanagawa 2300045, Japan. Balanced-size and long-size cloning of full-length, cap-trapped cDNAs into vectors of the novel λ-FLC family allows enhanced gene discovery rate and functional analysis. *Genomics* **77:** (1-2) 79.

Clark MD, Hennig S, Herwig R, Clifton SW, Marra MA, Lehrach H, Johnson SL. 2001. Max Planck Inst Mol Genet, DE-14195 Berlin, Germany. An oligonucleotide fingerprint normalized and expressed sequence tag characterized zebrafish cDNA library. *Genome Res* **11:** (9) 1594.

Hays DB, Skinner DZ. 2001. Kansas State Univ, Dept Entomol, Plant

Copyright © 2002 John Wiley & Sons, Ltd. 

Sci & Entomol Res Unit, Waters Hall, Manhattan, Ks 66506, USA. Development of an expressed sequence tag (EST) library for *Medicago sativa*. *Plant Sci* **161:** (3) 517.

Modrek B, Resch A, Grasso C, Lee C*. 2001. *UCLA, Dept Chem & Biochem, 611 Charles E Young Dr East, Los Angeles, Ca 90095, USA. Genome-wide detection of alternative splicing in expressed sequences of human genes. *Nucleic Acids Res* **29:** (13) 2850.

Smith EJ, Shi L, Prevost L, Drummond P, Ramlal S, Smith G, Pierce K, Foster J. 2001. Virginia Polytech Inst & State Univ, 3460 Litton Reaves Hall, Blacksburg, Va 24061, USA. Expressed sequence tags for the chicken genome from a normalized, ten-day-old white leghorn whole embryo cDNA library. II. Comparative DNA sequence analysis of guinea fowl, quail, and turkey genomes. *Poult Sci* **80:** (9) 1263.

Thomas SW, Rasmussen SW, Glaring MA, Rouster JA, Christiansen SK, Oliver RP. 2001. Carlsberg Lab, Dept Physiol, Gamle Carlsberg Vej 10, DK-2500 Copenhagen, Denmark. Gene identification in the obligate fungal pathogen *Blumeria graminis* by expressed sequence tag analysis. *Fungal Genet Biol* **33:** (33) 195.

Zeng CJ, Kouprina N, Zhu B, Cairo A, Hoek M, Cross G, Osoegawa K, Larionov V, De Jong P*. 2001. *Children's Hosp Oakland, BACPAC Resources, 747-52nd St, Oakland, Ca 94609, USA. Large-insert BAC/YAC libraries for selective re-isolation of genomic regions by homologous recombination in yeast. *Genomics* **77:** (1-2) 27.

### 9 Functional genomics

Delrue RM, Martinez-Lorenzo M, Lestrate P, Danese I, Bielarz V, Mertens P, De Bolle X, Tibor A, Gorvel JP, Letesson JJ. 2001. Univ Namur, Lab Immunol & Microbiol, URBM, rue Bruxelles 61, BE-5000 Namur, Belgium. Identification of *Brucella* spp, genes involved in intracellular trafficking. *Cell Microbiol* **3:** (7) 487.

Henrissat B, Coutinho PM, Davies GJ. 2001. CNRS UMR 6098, 31 Chemin Joseph Aiguier, FR-13402 Marseille 20, France. A census of carbohydrate-active enzymes in the genome of *Arabidopsis thaliana*. *Plant Mol Biol* **47:** (1-2) 55.

Huang L, Jacob RJ, Pegg SCH, Baldwin MA, Wang CC, Burlingame AL, Babbitt PC*. 2001. *Univ Calif, Dept Pharmaceut Chem, San Francisco, Ca 94143, USA. Functional assignment of the 20 S proteasome from *Trypanosoma brucei* using mass spectrometry and new bioinformatics approaches. *J Biol Chem* **276:** (30) 28327.

Kerkmann K, Lehming N*. 2001. *Max-Planck-Gesell, Max Delbruck Lab, Carl von Linne Weg 10, DE-50829 Cologne, Germany. Genome-wide expression analysis of a *Saccharomyces cerevisiae* strain deleted for the Tup1p-interacting protein Cdc73p. *Curr Genet* **39:** (5-6) 284.

Klee EW, Ekker SC, Ellis LBM*. 2001. *Univ Minnesota, Sch Med, Lab Med & Pathol, Mayo Mail Code 609, 420 SE Delaware St, Minneapolis, Mn 55455, USA. Target selection for *Danio redo* functional genomics. *Genesis* **30:** (3) 123.

Maizels RM, Blaxter ML, Scott AL. 2001. Univ Edinburgh, Inst Cell Anim & Populat Biol, West Mains Rd, Edinburgh EH9 3JT, Scotland. Immunological genomics of *Brugia malayi*: Filarial genes implicated in immune evasion and protective immunity. *Parasite Immunol* **23:** (7) 327.

Nizetic D. 2001. Univ London, Sch Pharm Pharmakol, Ctr Appl Mol Biol, 29-39 Brunswick Sq, London WC1N 1AX, England. Functional genomics of the Down syndrome (German, English Abstract). *Croat Med J* **42:** (4) 421.

Nomura H, Nishimori H, Yasoshima T*, Hata F, Sogahata K, Tanaka H, Nakajima F, Ikeda S, Kamiguchi K, Isomura H, Sato N, Denno R, Hirata K. 2001. *Sapporo Med Univ, Sch Med, Dept Surg 1, Chuo ku, S-1, W-16, Sapporo, Hokkaido 060 854, Japan. A novel experimental mouse model of peritoneal dissemination of human gastric cancer cells: Analysis of the mechanism of peritoneal dissemination using cDNA macroarrays. *Jpn J Cancer Res* **92:** (7) 748.

Oleksiak MF, Kolell KJ, Crawford DL*. 2001. *Univ Missouri, Div Mol Biol & Biochem, Kansas City, Mo 64110, USA. Utility of natural populations for microarray analyses: Isolation of genes necessary for functional genomic studies. *Mar Biotechnol* **3:** (Suppl 1) S203.

Sela-Buurlage MB, Budai-Hadrian O, Pan Q, Carmel-Goren L, Vunsch R, Zamir D, Fluhr R*. 2001. *Weizmann Inst Sci, Dept Plant Sci, POB 26, IL-76100 Rehovot, Israel. Genome-wide dissection of *Fusarium* resistance in tomato reveals multiple complex loci. *Mol Genet Genomics* **265:** (6) 1104.

Takahashi T, Shimoi H, Ito K. 2001. Kiku Masamune Sake Brewing Co Ltd, Gen Res Labs, Higashinada ku, 1-8-6 Uozaki Nishimachi, Kobe, Hyogo 658 0026, Japan. Identification of genes required for growth under ethanol stress using transposon mutagenesis in *Saccharomyces* cerevisiae. *Mol Genet Genomics* **265:** (6) 1112.

Tanaka A, Leung PSC, Kenny TP, Au Young J, Prindiville T, Coppel RL, Ansari AA, Gershwin ME*. 2001. *Univ Calif Davis, Sch Med, Dept Internal Med, Div Rheumatol Allergy & Clin Immunol, Davis, Ca 95616, USA. Genomic analysis of differentially expressed genes in liver and biliary epithelial cells of patients with primary biliary cirrhosis. *J Autoimmun* **17:** (1) 89.

Tax FE, Vernon DM*. 2001. *Whitman Coll, Dept Biol, Walla Walla, Wa 99362, USA. T-DNA-associated duplication/translocations in *Arabidopsis*. Implications for mutant analysis and functional genomics. *Plant Physiol* **126:** (4) 1527.

Throup JP, Zappacosta F, Lunsford RD, Annan RS, Carr SA, Lonsdale JT, Bryant AP, McDevitt D, Rosenberg M, Burnham MKR. 2001. GlaxoSmithKline Pharmaceut Res & Dev, Antinfect Res, Collegeville, Pa 19426, USA. The *srhSR* gene pair from *Staphylococcus aureus*: Genomic and proteomic approaches to the identification and characterization of gene function. *Biochemistry* **40:** (34) 10392.

Wang J, Zhang CT*. 2001. *Tianjin Univ, Dept Phys, CN-300072 Tianjin, Peoples Rep China. Identification of protein-coding genes in the genome of *Vibrio cholerae* with more than 98% accuracy using occurrence frequencies of single nucleotides. *Eur J Biochem* **268:** (15) 4261.

Whetten R, Sun YH, Zhang Y, Sederoff R. 2001. Nth Carolina State Univ, Forest Biotechnol Grp, 2500 Partners 2, Centennial Campus, Campus Box 7247, Raleigh, NC 27695, USA. Functional genomics and cell wall biosynthesis in loblolly pine. *Plant Mol Biol* **47:** (1-2) 275.

### 10 Transcriptomics

Allan E, Clayton CL, McLaren A, Wallace DM, Wren BW*. 2001. *Univ London, London Sch Hyg & Trop Med, Pathogen Mol Biol & Biochem Unit, Keppel St, London WC1E 7HT, England. Characterization of the low-pH responses of *Helicobacter pylori* using genomic DNA arrays. *Microbiology* **147:** (8) 2285.

Certa U, Seiler M, Padovan E, Spagnoli GC*. 2001. *Univ Basel, Dept Surg, Div Res, Basel, Switzerland. High density oligonucleotide array analysis of interferon-α2a sensitivity and transcriptional response in melanoma. *Br J Cancer* **85:** (1) 107.

Datson NA, Van der Perk J, De Kloet ER, Vreugdenhil E. 2001. Leiden Univ, Amsterdam Ctr Drug Res, Div Med Pharmacol, POB 9503, NL-2300 RA Leiden, The Netherlands. Expression profile of 30,000 genes in rat hippocampus using SAGE. *Hippocampus* **11:** (4) 430.

Evans CO, Young AN, Brown MR, Brat DJ, Parks JS, Neish AS, Oyesiku NM*. 2001. *Emory Univ, Sch Med, Lab Mol Neurosurg & Biotechnol, Dept Neurosurg, 136508 Clifton Rd NE, Atlanta, Ga 30322, USA. Novel patterns of gene expression in pituitary adenomas identified by complementary deoxyribonucleic acid microarrays and quantitative reverse transcription-polymerase chain reaction. *J Clin Endocrinol Metab* **86:** (7) 3097.

Kappe SHI, Gardner MJ, Brown SM, Ross J, Matuschewski K, Ribeiro JM, Adams JH, Quackenbush J, Cho J, Carucci DJ, Hoffman SL, Nussenzweig V. 2001. NYU, Sch Med, Dept Pathol, Kaplan Canc Ctr, Michael Heidelberger Div, New York, NY 10015, USA. Exploring the transcriptome of the malaria sporozoite stage. *Proc Natl Acad Sci U S A* **98:** (17) 9895.

Lapteva N, Ando Y, Nieda M, Hohjoh H, Okai M, Kikuchi A, Dymshits G, Ishikawa Y, Juji T, Tokunaga K*. 2001. *Univ Tokyo, Grad Sch Med, Dept Human Genet, Bunkyo ku, 7-3-1 Hongo, Tokyo 113 0033, Japan. Profiling of genes expressed in human monocytes and monocyte-derived dendritic cells using cDNA expression array. *Br J Haematol* **114:** (1) 191.

McCormick SM, Eskin SG, McIntire LV*, Teng CL, Lu CM, Russell CG, Chittur KK. 2001. *Rice Univ, Inst Biosci & Bioengn, Dept

Copyright © 2002 John Wiley & Sons, Ltd. 

Bioengn, 6100 Main St, Houston, Tx 77005, USA. DNA microarray reveals changes in gene expression of shear stressed human umbilical vein endothelial cells. *Proc Natl Acad Sci U S A* **98:** (16) 8955.

Nakajima T, Matsumoto K, Suto H, Tanaka K, Ebisawa M, Tomita H, Yuki K, Katsunuma T, Akasawa A, Hashida R, Sugita Y, Ogawa H, Ra C, Saito H*. 2001. *Natl Children's Med Res Ctr, Dept Allergy & Immunol, Setagaya ku, 3-35-31, Tokyo 154 8509, Japan. Gene expression screening of human mast cells and eosinophils using high-density oligonucleotide probe arrays: Abundant expression of major basic protein mast cells. *Blood* **98:** (4) 1127.

Saiura A, Mataki C, Murakami T, Umetani M, Wada Y, Kohro T, Aburatani H, Harihara Y, Hamakubo T, Yamaguchi T, Hasegawa T, Naito M, Makuudii M, Kodama T*. 2001. *Univ Tokyo, Adv Sci & Technol Res Ctr, Dept Mol Biol & Med, Meguro ku, 4-6-1 Komaba, Tokyo 153 8904, Japan. A comparison of gene expression in murine cardiac allografts and isografts by means of DNA microarray analysis. *Transplantation* **72:** (2) 320.

Seo J, Kim M, Kim J*. 2000. *Kyungpook Natl Univ, Sch Med, Dept Immunol, Deagu 700422, Rep Sth Korea. Identification of novel genes differentially expressed in PMA-induced HL-60 cells using cDNA microarrays. *Mol Cells* **10:** (6) 733.

Tabaska JE, Davuluri RV, Zhang MQ*. 2001. *Cold Spring, Harbor Lab, POB 100, Cold Spring Harbor, NY 11724, USA. Identifying the 3′-terminal exon in human DNA. *Bioinformatics* **17:** (7) 602.

Wassarman KM, Repoila F, Rosenow C, Storz G, Gottesman S*. 2001. *NIH/NICHHD, Cell Biol & Metab Branch, Bethesda, Md 20892, USA. Identification of novel small RNAs using comparative genomics and microarrays. *Gene Dev* **15:** (13) 1637.

Zheng M, Wang X, Templeton LJ, Smulski DR, La Rossa RA, Storz G*. 2001. *NIH/NICHHD, Cell Biol & Metab Branch, Bldg 18T, Room 101, 18 Library Dr, MSC 5430, Bethesda, Md 20892, USA. DNA microarray-mediated transcriptional profiling of the *Escherichia coli* response to hydrogen peroxide. *J Bacteriol* **183:** (15) 4562.

### 11 Proteomics

Adams P, Fowler R, Kinsella N, Howell G, Farris M, Coote P, O'Connor CD*. 2001. *Univ Southampton, Sch Biol Sci, Div Biochem & Mol Biol, Bassett Crescent East, Southampton SO16 7PX, England. Proteomic detection of PhoPQ- and acid-mediated repression of *Salmonella* motility. *Proteomics* **1:** (4) 597.

Allen NPC, Huang L, Burlingame A, Rexach M*. 2001. *Stanford Univ, Dept Biol Sci, Stanford, Ca 94305, USA. Proteomic analysis of nucleoporin interacting proteins. *J Biol Chem* **276:** (31) 29268.

Antelmann H, Tjalsma H, Voigt B, Ohlmeier S, Bron S, Van Dijl JM*, Hecker M. 2001. *Univ Groningen, Dept Pharmaceut Biol, Groningen, The Netherlands. A proteomic view on genome-based signal peptide predictions. *Genome Res* **11:** (9) 1484.

Belghazi M, Bathany K, Hountondji C, Grandier-Vazeilla X, Manon S, Schmitter JM*. 2001. *Univ Bordeaux 1, CNRS UMR 5472, Lab Phys & Tox Chim Syst Nat, FR-33405 Talence, France. Analysis of protein sequences and protein complexes by matrix-assisted laser desorption/ionization mass spectrometry. *Proteomics* **1:** (8) 946.

Bruneau JM, Magnin T, Tagat E, Legrand R, Bernard M, Diaquin M, Fudali C, Latge JP. 2001. Aventis Hoechst Marion Roussel, Dept Biochem, Infect Dis Grp, 102 Route Noisy, FR-93235 Romainville, France. Proteome analysis of *Aspergillus fumigatus* identifies glycosylphosphatidylinositol-anchored proteins associated to the cell wall biosynthesis. *Electrophoresis* **22:** (13) 2812.

Choi JS, Kim DS, Lee J, Kim SJ, Kim SI, Kim YH, Hong J, Yoo JS, Suh KH, Park YM. 2000. Korea Basic Sci Inst, Biomol Res Team, Taejon 305333, Rep Sth Korea. Proteome analysis of light-induced proteins in *Synechocystis* sp PCC 6803: Identification of proteins separated by 2D-PAGE using N-terminal sequencing and MALDI-TOF MS. *Mol Cells* **10:** (6) 705.

Covert BA, Spencer JS, Orme IM, Belisle JT*. 2001. *Colorado State Univ, Dept Microbiol, Mycobacteria Res Labs, Fort Collins, Co 80523, USA. The application of proteomics in defining the T-cell antigens of *Mycobacterium tuberculosis*. *Proteomics* **1:** (4) 574.

Cunsolo V, Foti S, Saletti R, Ceraulo L*, Di Stefano V. 2001. *Univ Palermo, Dipt Chim & Tecnol Farmaceut, via Archirafi 32, IT-90123 Palermo, Italy. Detection and localisation of disulphide bonds in a synthetic peptide reproducing the sequence 1-30 of Par j 1.0101 by electrospray ionisation mass spectrometry. *Proteomics* **1:** (8) 1043.

Evers S, Di Padova K, Meyer M, Langen H, Fountoulakis M, Keck W, Gray CP. 2001. F Hoffmann La Roche & Co Ltd, Biol Technol, Bldg 93-5-58, CH-4070 Basel, Switzerland. Mechanism-related changes in the gene transcription and protein synthesis patterns of *Haemophilus influenzae* after treatment with transcriptional and translational inhibitors. *Proteomics* **1:** (4) 522.

Forbes AJ, Mazur MT, Patel HM, Walsh CT, Kelleher NL*. 2001. *Univ Illinois, Dept Chem, 600 Sth Matthews Ave, Urbana, Il 61801, USA. Toward efficient analysis of >70 kDa proteins with 100% sequence coverage. *Proteomics* **1:** (8) 927.

Giometti CS, Reich CI, Tollaksen SL, Babnigg G, Lim H, Yates JR, Olsen GJ. 2001. Argonne Natl Lab, Div Biosci, 9700 Sth Cass Ave, Bldg 202, Room B117, Argonne, Il 60439, USA. Structural modification of *Methanococcus jannaschii* flagellin proteins revealed by proteome analysis. *Proteomics* **1:** (8) 1033.

Goldfarb M. 2001. Anatek-EP, 17 Bishop St, Portland, Me 04103, USA. Analysis of antibody to milk proteins in HIV positive/negative sera using two-dimensional electrophoresis, Western blot and immunoassay. *Proteomics* **1:** (5) 721.

Greco A, Bienvenut W, Sanchez JC, Kindbeiter K, Hochstrasser D, Madjar JJ, Diaz JJ*. 2001. *Med Lyon RTH Laennec, INSERM U369, 7 rue Guillaume Paradin, FR-69372 Lyon 08, France. Identification of ribosome-associated viral and cellular basic proteins during the course of infection with herpes simplex virus type 1. *Proteomics* **1:** (4) 545.

Guillaume E, Pineau C*, Evrard B, Dupaix A, Moertz E, Sanchez JC, Hochstrasser DF, Jegou B. 2001. *Univ Rennes 1, GERM-INSERM U435, Campus Beaulieu, FR-35042 Rennes, France. Cellular distribution of translationally controlled tumor protein in rat and human testes. *Proteomics* **1:** (7) 880.

Hernychova L, Stulik J*, Halada P, Macela A, Kroca M, Johansson T, Malina M. 2001. *Purkyne Milit Med Acad, Inst Radiobiol & Immunol, Trebesska 1575, CZ-50001 Hradec Kralove, Czech Republic. Construction of a *Francisella tularensis* two-dimensional electrophoresis protein database. *Proteomics* **1:** (4) 508.

Hoffmann P, Ji H, Moritz RL, Connolly LM, Frecklington DF, Layton MJ, Eddes JS, Simpson RJ*. 2001. *Royal Melbourne Hosp, Walter & Eliza Hall Inst Med Res, Ludwig Inst Canc Res, Parkville, Vic 3050, Australia. Continuous free-flow electrophoresis separation of cytosolic proteins from the human colon carcinoma cell line LIM 1215: A non two-dimensional gel electrophoresis-based proteome analysis strategy. *Proteomics* **1:** (7) 807.

Isharc H, Watling D, Kerr IM. 2001. ICRF, BRM 509, 44 Lincoln Inn Fields, London WC2A 3PX, England. Phosphotyrosine profiling to identify novel components of interferon and interleukin 6-family cytokine signalling. *Proteomics* **1:** (6) 767.

Jungblut PR, Muller EC, Mattow J, Kaufmann SHE. 2001. Max-Planck-Inst Infect Biol, Core Facil Protein Anal, Schumannstr 21-22, DE-10117 Berlin, Germany. Proteomics reveals open reading frames in *Mycobacterium tuberculosis* H37Rv not predicted by genomics. *Infect Immun* **69:** (9) 5905.

Kernec F, Unlu M, Labeikovsky W, Minden JS, Koretsky AP*. 2001. *NINCDS, Lab Funct & Mol Imaging, Rm 36-5805, MSC 4159, 36 Convent Dr, Bethesda, Md 20892, USA. Changes in the mitochondrial proteome from mouse hearts deficient in creatine kinase. *Physiol Genomics* **6:** (2) 117.

Klade CS, Voss T, Krystek E, Ahorn H, Zatloukal K, Pummer K, Adolf GR. 2001. Intercell GmbH, Rennweg 95B, AU-1030 Vienna, Austria. Identification of tumor antigens in renal cell carcinoma by serological proteome analysis. *Proteomics* **1:** (7) 890.

Kovarova H, Necasova R, Porkertova S, Radzioch D, Macela A. 2001. Purkyne Milit Med Acad, Inst Radiobiol & Immunol, Trebesska Str 1575, CZ-50001 Hradec Kralove, Czech Republic. Natural resistance to intracellular pathogens: Modulation of macrophage signal transduction related to the expression of the *Bcg* locus. *Proteomics* **1:** (4) 587.

Lanne B, Potthast F, Hoglund A, Von Lowenhielm HB, Nystrom AC, Nilsson F, Dahllof B. 2001. AstraZeneca R&D, Res Area CV & GI, SE-43183 Molndal, Sweden. Thiourea enhances mapping of the proteome from murine white adipose tissue. *Proteomics* **1:** (7) 819.

Copyright © 2002 John Wiley & Sons, Ltd. 

Lawrie LC, Curran S, McLeod HL, Fothergill JE, Murray GI*. 2001. *Univ Aberdeen, Dept Pathol, Aberdeen AB9 1FX, Scotland. Application of laser capture microdissection and proteomics in colon cancer. *J Clin Pathol-Mol Pathol* **54:** (4) 253.

Lefkovits I, Kettman JR, Frey JR. 2001. Basel Inst Immunol, Grenzacherstr 487, CH-4005 Basel, Switzerland. Proteomic analysis of rare molecular species of transiated polypeptides from a mouse fetal thymus cDNA library. *Proteomics* **1:** (4) 560.

Leung KY, Wait R, Welson SY, Yan YX, Abranham DJ, Black CM, Pearson JD, Dunn MJ. 2001. Harefield Hosp, Imperial Coll, Sch Med, Natl Heart & Lung Inst Heart Sci Ctr, Harefield UB9 6JH, England. A reference map of human lung MRC-5 fibroblast proteins using immobilized pH gradient-isoelectric focusing-based two-dimensional electrophoresis. *Proteomics* **1:** (6) 787.

Li J, Tan C, Xiang Q, Zhang XM, Ma J, Wang JR, Yang JB, Li WF, Shen SR, Liang SP*, Li G. 2001. *Hunan Normal University, College Life Science, CN-410006 Changsha, Hunan, Peoples Rep China. Proteomic detection of changes in protein synthesis induced by *NGX6* transfected in human nasopharyngeal carcinoma cells. *J Protein Chem* **20:** (3) 265.

Lim DB, Hains P, Walsh B, Bergquist P, Nevalainen H*. 2001. *MacQuarie Univ, Dept Biol Sci, Sydney, NSW 2109, Australia. Proteins associated with the cell envelope of *Trichoderma reesei*: A proteomic approach. *Proteomics* **1:** (7) 899.

Lock RA, Cordwell SJ, Coombs GW, Walsh BJ, Forbes GM. 2001. Royal Perth Hosp, Dept Microbiol & Infect Dis, Perth, WA 6000, Australia. Proteome analysis of *Helicobacter pylori*: Major proteins of type strain NCTC 11637. *Pathology* **33:** (3) 365.

Loo RRO, Cavalcoli JD, Van Bogelen RA, Mitchell C, Loo JA, Moldover B, Andrews PC. 2001. Pfizer Global Res & Dev, 2800 Plymouth Rd, Ann Arbor, Mi 48105, USA. Virtual 2-D gel electrophoresis: Visualization and analysis of the *E. coli* proteome by mass spectrometry. *Anal Chem* **73:** (17) 4063.

Mattow J, Jungblut PR, Muller EC, Kaufmann SHE. 2001. Max-Planck-Inst Infect Biol, Dept Immunol, Schumannstr 21-22, DE-10117 Berlin, Germany. Identification of acidic, low molecular mass proteins of *Mycobacterium tuberculosis* strain H37Rv by matrix-assisted laser desorption/ionization and electrospray ionization mass spectrometry. *Proteomics* **1:** (4) 494.

McAtee CP, Hoffman PS, Berg DE. 2001. Bristol Myers Squbb Co, Pharmaceut Res Inst, Dept Appl Genomics, POB 5400, Princeton, NJ 08534, USA. Identification of differentially regulated proteins in metronidozole resistant *Helicobacter pylori* by proteome techniques. *Proteomics* **1:** (4) 516.

Mills PB, Mills K, Johnson AW, Clayton PT, Winchester BG*. 2001. *Inst Child Hlth, Biochem Endocrinol & Metab Unit, Biochem Dept, 30 Guilford St, London WC1N 1EH, England. Analysis by matrix assisted laser desorption/ionisation-time of flight mass spectrometry of the post-translational modifications of $\alpha_1$-antitrypsin isoforms separated by two-dimensional polyacrylamide gel electrophoresis. *Proteomics* **1:** (6) 778.

Nawrocki A, Fey SJ, Goffeau A, Roepstorff P, Larsen PM*. 2001. *Univ Sthn Denmark, Int Sci Pk Odense, Ctr Proteome Anal Life Sci, Forskerparken 108, DK-5230 Odense M, Denmark. The effects of transcription regulating genes *PDR1, pdr1-3* and *PDR3* in pleiotropic drug resistance. *Proteomics* **1:** (8) 1022.

Ou K, Seow TK, Liang RCMY, Ong SE, Chung MCM. 2001. Natl Univ Singapore, Ctr Bioproc Technol, Level 5 10 Med Dr, SG-117597 Singapore, Rep Singapore. Proteome analysis of a human heptocellular carcinoma cell line, HCC-M: An update. *Electrophoresis* **22:** (13) 2804.

Phadke ND, Molloy MP, Steinhoff SA, Ulintz PJ, Andrews PC, Maddock JR*. 2001. *Univ Michigan, Dept Biol, 830 Nth Univ, Ann Arbor, Mi 48109, USA. Analysis of the outer membrane proteome of *Caulobacter crescentus* by two-dimensional electrophoresis and mass spectrometry. *Proteomics* **1:** (5) 705.

Pitarch A, Diez Orejas R, Molero G, Pardo M, Sanchez M, Gill C*, Nombela C. 2001. *Univ Complutense, Fac Farm, Dept Microbiol II, ES-28040 Madrid, Spain. Analysis of the serologic response to systemic *Candida albicans* infection in a murine model. *Proteomics* **1:** (4) 550.

Sanchez-Fernandez R, Davies TGE, Coleman JOD, Rea PA*. 2001. *Univ Penn, Dept Biol, Inst Plant Sci, Philadelphia, Pa 19104, USA. The *Arabidopsis thaliana* ABC protein superfamily, a complete inventory. *J Biol Chem* **276:** (32) 30231.

Steyn B, Oosthuizen MC, MacDonald R, Theron J, Brozel VS*. 2001. *Univ Pretoria, Dept Microbiol & Plant Pathol, Lab Biofilm Physiol, ZA-0002 Pretoria, South Africa. The use of glass wool as an attachment surface for studying phenotypic changes in *Pseudomonas aeruginosa* biofilms by two-dimensional gel electrophoresis. *Proteomics* **1:** (7) 871.

Suzuki T, Terasaki M, Takemoto-Hori C, Hanada T, Ueda T, Wada A, Watanabe K. 2001. Univ Tokyo, Grad Sch Frontier Sci, Dept Integrated Biosci, 5-1-5 Kashiwanoha, Kashiwa, Chiba 277 8562, Japan. Proteomic analysis of the mammalian mitochondrial ribosome: Identification of protein components in the 28 S small subunit. *J Biol Chem* **276:** (35) 33181.

Thiede B, Dimmler C, Siejak F, Rudel T*. 2001. *Max-Planck-Inst Infektionsbiol, Abt Mol Biol, Schumannstr 21-22, DE-10117 Berlin, Germany. Predominant identification of RNA-binding proteins in Fas-induced apoptosis by proteome analysis. *J Biol Chem* **276:** (28) 26044.

Von Haller PD, Donohoe S, Goodlett DR, Aebersold R, Watts JD*. 2001. *Inst Syst Biol, 4225 Roosevelt Way, Suite 200, Seattle, Wa 98105, USA. Mass spectrometric characterization of proteins extracted from Jurkat T-cell detergent-resistant membrane domains. *Proteomics* **1:** (8) 1010.

Vosseller K, Wells L, Hart GW*. 2001. *Johns Hopkins Sch Med, Dept Biol Chem, 725 Nth Wolfe St, Baltimore, Md 21205, USA. Nucleocytoplasmic *O*-glycosylation: *O*-GlcNAc and functional proteomics. *Biochimie* **83:** (7) 575.

Zhou XH, Gonnet H, Hallett M, Munchbach M, Folkers G, James P*. 2001. *Alte Landstr 83, CH-8803 Ruschlikon, Switzerland. Cell fingerprinting: An approach to classifying cells according to mass profiles of digests of protein extracts. *Proteomics* **1:** (5) 683.

Ziebandt AK, Weber H, Rudolph J, Schmid R, Hoper D, Engelmann S, Hecker M*. 2001. *Inst Mikrobiol & Mol Biol, Jahnstr 15, DE-17487 Greifswald, Germany. Extracellular proteins of *Staphylococcus aureus* and the role of SarA and $\delta^B$. *Proteomics* **1:** (4) 480.

### 12 Protein structural genomics

Cook DJ, Holder LB, Su SB, Maglothin R, Jonyer I. 2001. Univ Texas, Dept Comp Sci & Engn, POB 19015, Arlington, Tx 76019, USA. Structural mining of molecular biology data. *IEEE Eng Med Biol Mag* **20:** (4) 67.

Liu RX, Blackwell TW, States DJ*. 2001. *Washington Univ, Sch Med, Ctr Computat Biol, 700 Sth Euclid Ave, St Louis, Mo 63110, USA. Conformational model for binding site recognition by the *E. coli* MetJ transcription factor. *Bioinformatics* **17:** (7) 622.

Moller S, Croning M D R, Apweiler R. 2001. EMBL, Outstn European Bioinformatics Inst, Wellcome Trust, Genome Campus, Cambridge CB10 1SD, England. Evaluation of methods for the prediction of membrane spanning regions. *Bioinformatics* **17:** (7) 646.

Murvai J, Vlahovicek K, Szepesvari C, Pongor S*. 2001. *Int Ctr Genet Engn & Biotechnol, Protein Struct & Funct Grp, IT-34012 Trieste, Italy. Prediction of protein functional domains from sequences using artificial neural networks. *Genome Res* **11:** (8) 1410.

### 13 Metabolomics

Ter Kuile BH, Westerhoff HV. 2001. Leiden Inst Chem, POB 9502, NL-2300 RA Leiden, The Netherlands. Transcriptome meets metabolome: Hierarchical and metabolic regulation of the glycolytic pathway. *FEBS Lett* **500:** (3) 169.

Tsoka S, Ouzounis CA*. 2001. *Eur Mol Biol Lab, Eur Bioinformat Inst, Res Programme, Computat Genomics Grp, Cambridge CB10 1SD, England. Functional versatility and molecular diversity of the metabolic map of *Escherichia coli*. *Genome Res* **11:** (9) 1503.

Copyright © 2002 John Wiley & Sons, Ltd. 

### 14 Genomic approaches to development

Altmann CR, Bell E, Sczyrba A, Pun J, Bekiranov S, Gaasterland T, Brivanlou AH*. 2001. *Rockefeller Univ, Lab Mol Vertebrate Embryol, 1230 York Ave, New York, NY 10021, USA. Microarray-based analysis of early development in *Xenopus laevis*. *Dev Biol* **236:** (1) 64.

Bommert P, Werr W*. 2001. *Univ Cologne, Inst Entwickungsbiol, DE-50923 Cologne, Germany. Gene expression patterns in the maize caryopsis: Clues to decisions in embryo and endosperm development. *Gene* **271:** (2) 131.

Boyes DC, Zayed AM, Ascenzi R, McCaskill AJ, Hoffman NE, Davis KR*, Gorlach J. 2001. *Paradigm Genet Inc, Dept Plant Res, Res Triangle Park, NC 27709, USA. Growth stage-based phenotypic analysis of *Arabidopsis*: A model for high throughput functional genomics in plants. *Plant Cell* **13:** (7) 1499.

Furlong EEM, Andersen EC, Null B, White KP, Scott MP*. 2001. *Stanford Univ, Sch Med, Howard Hughes Med Inst, Dept Dev Biol, Beckman Ctr, 279 Campus Dr, Stanford, Ca 94305, USA. Patterns of gene expression during *Drosophila* mesoderm development. *Science* **293:** (5535) 1629.

Lian Z, Wang L, Yamaga S, Bonds W, Beazer-Barclay Y, Kluger Y, Gerstein M, Newburger PE, Berliner N, Weissman SM*. 2001. *Yale Univ, Sch Med, Dept Genet, Boyer Ctr Mol Med, 295 Congress Ave, New Haven, Ct 06536, USA. Genomic and proteomic analysis of the myeloid differentiation program. *Blood* **98:** (3) 513.

Mody M, Cao YX, Cui ZZ, Tay KY, Shyong A, Shimizu E, Pham K, Schultz P, Welsh D, Tsien JZ*. 2001. *Princeton Univ, Dept Mol Biol, Washington Rd, Princeton, NJ 08544, USA. Genome-wide gene expression profiles of the developing mouse hippocampus. *Proc Natl Acad Sci U S A* **98:** (15) 8862.

Vohradsky J, Ramsden JJ*. 2001. *Hochstr 51, CH-4053 Basel, Switzerland. Genome resource utilization during prokaryotic development. *FASEB J* **15:** (9) U6.

### 15 Technological advances

Alexandre I, Hamels S, Dufour S, Collet J, Zammatteo N, De Longueville F, Gala JL, Remacle J. 2001. Fac Univ Notre Dame Paix, Lab Biochim Cellulaire, 61 rue Bruxelles, BE-5000 Namur, Belgium. Colorimetric silver detection of DNA microarrays. *Anal Biochem* **295:** (1) 1.

Benters R, Niemeyer CM*, Wohrle D. 2001. *Univ Bremen, Inst Organ & Macromol Chem, FB2-UFT, POB 33.04.40, DE-28334 Bremen, Germany. Dendrimer-activated-solid supports for nucleic acid and protein microarrays. *Chembiochem* **2:** (9) 686.

Brown CS, Goodwin PC, Sorger PK*. 2001. *MIT BioMicro Ctr, Dept Biol, 77 Massachusetts Ave, Room 68-371, Cambridge, Ma 02139, USA. Image metrics in the statistical analysis of DNA microarray data. *Proc Natl Acad Sci U S A* **98:** (16) 8944.

Buzas Z, Chang HT, Vieira NE, Yergey AL, Stastna M, Chrambach A*. 2001. *NIH/NICHHD, Macromol Anal Sect, Bldg 10, Room 9D50, Bethesda, Md 20892, USA. Direct vertical electroelution of protein from a PhastSystem band for mass spectrometric identification at the levels of a few picomoles. *Proteomics* **1:** (5) 691.

Choudhary JS, Blackstock WP, Creasy DM, Cottrell JS*. 2001. *Matrix Sci Ltd, 30 Harcourt St, London W1H 4HT, England. Interrogating the human genome using uninterpreted mass spectrometry data. *Proteomics* **1:** (5) 651.

Compagnini A, Cunsolo V, Foti S*, Saletti R. 2001. *Univ Catania, Dipt Sci Chim, Viale A Doria 6, IT-95125 Catania, Italy. Improved accuracy in the matrix-assisted laser desorption/ionization-mass spectrometry determination of the molecular mass of cyanogen bromide fragments of proteins by post-cleavage reaction with tris(hydroxymethyl)aminomethane. *Proteomics* **1:** (8) 967.

Conrad CC, Choi JG, Malakowsky CA, Talent JM, Dai R, Marshall P, Gracy RW*. 2001. *Off Res & Biotechnol, Room 806, 3500 Camp Bowie Blvd, Fort Worth, Tx 76107, USA. Identification of protein carbonyls after two-dimensional electrophoresis. *Proteomics* **1:** (7) 829.

Doucette A, Li L*. 2001. *Univ Alberta, Dept Chem, Edmonton, Alberta, Canada T6G 292. Investigation of the applicability of a sequential digestion protocol using trypsin and leucine aminopeptidase M for protein identification by matrix-assisted laser desorption/ionization time of flight mass spectrometry. *Proteomics* **1:** (8) 987.

Dunlop KY, Li L*. 2001. *Univ Alberta, Dept Chem, Edmonton, Alberta, Canada T6G 2G2. Automated mass analysis of low-molecular-mass bacterial proteome by liquid chromatography-electrospray ionization mass spectrometry. *J Chromatogr A* **925:** (1-2) 123.

Fernandez PL, Nayach I, Fernandez E, Fresno L, Palacin A, Farre X, Campo E, Cardesa A. 2001. Univ Barcelona, Hosp Clin Barcelona, Dept Anat & Pathol, ES-08036 Barcelona, Spain. Tissue macroarrays (“microchips”) for gene expression analysis. *Virchows Archiv* **438:** (6) 591.

He MY, Taussig MJ. 2001. Babraham Inst, Technol Res Grp, Cambridge CB2 4AT, England. Single step generation of protein arrays from DNA by cell-free expression and *in situ* immobilisation (PISA method). *Nucleic Acids Res* **29:** (15) U18.

Huang RP. 2001. Emory Univ, Sch Med, Dept Gynecol & Obstet, Div Res, 1639 Pierce Dr, Room 4219, Atlanta, Ga 30322, USA. Detection of multiple proteins in an antibody-based protein microarray system. *J Immunol Methods* **255:** (1-2) 1.

Jiang Y, Lee CS*. 2001. *Univ Maryland, Dept Chem & Biochem, College Park, Md 20742, USA. On-line coupling of micro-enzyme reactor with micro-membrane chromatography for protein digestion, peptide separation, and protein identification using electrospray ionization mass spectrometry. *J Chromatogr A* **924:** (1-2) 315.

Kuster B, Mortensen P, Andersen JS, Mann M*. 2001. *Univ Sthn Denmark, Protein Interact Lab, Campusvej 55, DK-5230 Odense M, Denmark. Mass spectrometry allows direct identification of proteins in large genomes. *Proteomics* **1:** (5) 641.

Layfield R, Tooth D, Landon M, Dawson S, Mayer J, Alban A. 2001. Univ Nottingham Med Sch, Sch Biomed Sci, Queen’s Med Ctr, Nottingham NG7 2UH, England. Purification of poly-ubiquitinated proteins by S5a-affinity chromatography. *Proteomics* **1:** (6) 773.

Li JJ, Tremblay TL, Wang C, Attiya S, Harrison DJ, Thibault P*. 2001. *Inst Biol Sci, 100 Sussex Dr, Ottawa, Ontario, Canada K1A 0R6. Integrated system for high-throughput protein identification using a microfabricated device coupled to capillary electrophoresis/nanoelectrospray mass spectrometry. *Proteomics* **1:** (8) 975.

Ma Y, Lu Y, Zeng H, Ron D, Mo W, Neubert TA*. 2001. *Univ Sch Med, Dept Pharmacol, Skirball Inst Biomolec Med, New York, NY 10016, USA. Characterization of phosphopeptides from protein digests using matrix-assisted laser desorption/ionization time- of-flight mass spectrometry and nanoelectrospray quadrupole time- of-flight mass spectrometry. *Rapid Commun Mass Spectrom* **15:** (18) 1693.

Medintz IL, Paegel BM, Mathies RA*. 2001. *Univ Calif, Dept Chem, MS 1460, Berkeley, Ca 94720, USA. Microfabricated capillary array electrophoresis DNA analysis systems. *J Chromatogr A* **924:** (1-2) 265.

Meiyanto E, Mineno J, Ishida N, Takeya T*. 2001. *Nara Inst Sci & Technol, Grad Sch Biol Sci, Nara 630 0101, Japan. Application of fluorescently labeled poly(dU) for gene expression profiling on cDNA microarrays. *Biotechniques* **31:** (2) 406.

Poutanen M, Salusjarvi L, Ruohonen L, Penttila M, Kalkkinen N. 2001. Univ Helsinki, Inst Biotechnol, Protein Chem Lab, PO Box 56, Viikinkaari 9, FI-00014 Helsinki, Finland. Use of matrix-assisted laser desorption/ionization time-of-flight mass mapping and nanospray liquid chromatography/electrospray ionization tandem mass spectrometry sequence tag analysis for high sensitivity identification of yeast proteins separated by two-dimensional gel electrophoresis. *Rapid Commun Mass Spectrom* **15:** (18) 1685.

Rabilloud T, Strub JM, Luche S, Van Dorsselaer A, Lunardi J. 2001. DBMS-BECP, CEA-Grenoble, Lab Bioenerget Cellulaire & Pathol, 17 rue Martyrs, FR-38054 Grenoble 9, France. Comparison between Sypro Ruby and ruthenium II tris (bathophenanthroline disulfonate) as fluorescent stains for protein detection in gels. *Proteomics* **1:** (5) 699.

Riggs L, Sioma C, Regnier FE*. 2001. *Purdue Univ, Dept Chem, West Lafayette, In 47907, USA. Automated signature peptide approach for proteomics. *J Chromatogr A* **924:** (1-2) 359.

Sinha P, Poland J, Schnolzer M, Rabilloud T. 2001. Univ Klin Charite, Inst Lab & Med Pathobiochem, Campus Charite Mitte, Schumannstr 20-21, DE-10117 Berlin, Germany. A new silver staining apparatus

Copyright © 2002 John Wiley & Sons, Ltd. 

and procedure for matrix-assisted laser desorption/ionization-time of flight analysis of proteins after two-dimensional electrophoresis. *Proteomics* **1:** (7) 835.

Steinberg TH, Top KPO, Berggren KN, Kemper C, Jones L, Diwu ZJ, Haugland RP, Patton WF*. 2001. *Molecular Probes Inc, Proteomics Sect, 4849 Pitchford Ave, Eugene, Or 97402, USA. Rapid and simple single nanogram detection of glycoproteins in polyacrylamide gels and on electroblots. *Proteomics* **1:** (7) 841.

Stensballe A, Jensen ON*. 2001. *Odense Univ, Univ Sthn Denmark, Dept Biochem & Mol Biol, Campusvej 55, DK-5230 Odense, Denmark. Simplified sample preparation method for protein identification by matrix-assisted laser desorption/ionization mass spectrometry: In-gel digestion on the probe surface. *Proteomics* **1:** (8) 955.

Taylor E, Cogdell D, Coombes K, Hu L, Ramdas L, Tabor A, Hamilton S, Zhang W*. 2001. *Univ Texas, MD Anderson Canc Ctr, Dept Pathol, Canc Genomics Lab, 1515 Holcombe Blvd, Houston, Tx 77030, USA. Sequence verification as quality-control step for production of cDNA microarrays. *Biotechniques* **31:** (1) 62.

Uttenweiler-Joseph S, Neubauer G, Christoforidis A, Zerial M, Wilm M*. 2001. *Eur Mol Biol Lab, Meyerhofstr 1, DE-69117 Heidelberg, Germany. Automated *de novo* sequencing of proteins using the differential scanning techniques. *Proteomics* **1:** (5) 668.

Veeser S, Dunn MJ, Yang GZ*. 2001. *Univ London, Imperial Coll Sci Technol & Med, Dept Comp, Royal Soc Wolfson Fdn Med Image Comp Lab, London SW7 2BZ, England. Multiresolution image registration for two-dimensional gel electrophoresis. *Proteomics* **1:** (7) 856.

Wall DB, Kachman MT, Gong SS, Parus SJ, Long MW, Lubman DM*. 2001. *Univ Michigan, Dept Chem, Ann Arbor, Mi 48109, USA. Isoelectric focusing nonporous silica reversed-phase high-performance liquid chromatography/electrospray ionization time-of-flight mass spectrometry: A three-dimensional liquid-phase protein separation method as applied to the human erythroleukemia cell-line. *Rapid Commun Mass Spectrom* **15:** (18) 1649.

Wang SH, Regnier FE*. 2001. *Purdue Univ, Dept Chem, West Lafayette, In 47907, USA. Proteomics based on selecting and quantifying cysteine containing peptides by covalent chromatography. *J Chromatogr A* **924:** (1-2) 345.

Zhang ZL, Smith DL, Smith JB*. 2001. *Univ Nebraska, Dept Chem, Lincoln, Ne 68588, USA. Multiple separations facilitate identification of protein variants by mass spectrometry. *Proteomics* **1:** (8) 1001.

### 16 Bioinformatics

Ashburner M, Ball CA, Blake JA, Butler H, Cherry JM, Corradi J, Dolinski K, Eppig JT, Harris M, Hill DP, Lewis S, Marshall B, Mungall C, Reiser L, Rhee S, Richardson JE, Richter J, Ringwald M, Rubin GM, Sherlock G, Yoon J. 2001. Address not supplied. Creating the gene ontology resource: Design and implementation. *Genome Res* **11:** (8) 1425.

Berrar D, Dubitzky W, Solinas-Toldo S, Bulashevska S, Granzow M, Conrad C, Kalla J, Lichter P, Eils R. 2001. German Canc Res Ctr, Intelligent Bioinformat Syst Div, Neuenheimer Feld 280, DE-69120 Heidelberg, Germany. A database system for comparative genomic hybridization analysis. *IEEE Eng Med Biol Mag* **20:** (4) 75.

Bertone P, Gerstein M*. 2001. *Yale Univ, Dept Mol Biophys & Biochem, New Haven, Ct 06520, USA. Integrative data mining: The new direction in bioinformatics. *IEEE Eng Med Biol Mag* **20:** (4) 33.

Bertone P, Kluger Y, Lan N, Zheng DY, Christendat D, Yee A, Edwards AM, Arrowsmith CH, Montelione GT, Gerstein M*. 2001. *Address as above. SPINE: An integrated tracking database and data mining approach for identifying feasible targets in high-throughput structural proteomics. *Nucleic Acids Res* **29:** (13) 2884.

Chen X, Kwong S*, Li M. 2001. *City Univ Hong Kong, Dept Comp Sci, 83 Tatchee Ave, Kowloon, Hong Kong, Peoples Rep China. A compression algorithm for DNA sequences. *IEEE Eng Med Biol Mag* **20:** (4) 61.

Comander J, Weber GM, Gimbrone MA, Garcia-Cardena G*. 2001. *Brigham & Womens Hosp, Dept Pathol, Div Vasc Res, Ctr Excellence Vasc Biol, Boston, Ma 02115, USA. Argus: A new database system for web-based analysis of multiple microarray data sets. *Genome Res* **11:** (9) 1603.

Dalevi D, Andersson SGE*. 2001. *Uppsala Univ, Dept Mol Evolut, Linnaeur Ctr Bioinformatics, Norbyvagen 18C, SE-75236 Uppsala, Sweden. Discovering the dynamics of microbial genomes. *IEEE Eng Med Biol Mag* **20:** (4) 55.

Devos D, Valencia A. 2001. CNB CSIC, Protein Design Grp, ES-28049 Madrid, Spain. Intrinsic errors in genome annotation. *Trends Genet* **17:** (8) 429.

Drummond A, Strimmer K*. 2001. *Univ Oxford, Dept Zool, Sth Parks Rd, Oxford OX1 3PS, England. PAL: An object-oriented programming library for molecualr evolution and phylogenetics. *Bioinformatics* **17:** (7) 662.

Eckman BA, Kosky AS, Laroco LA. 2001. GlaxoSmithKline, Dept Bioinformat, King of Prussia, Pa, USA. Extending traditional query-based integration approaches for functional characterization of post-genomic data. *Bioinformatics* **17:** (7) 587.

Ewing RM, Cherry JM. 2001. Carnegie Inst Washington, Dept Plant Biol, 260 Panama St, Stanford, Ca 94305, USA. Visualization of expression clusters using Sammon's non-linear mapping. *Bioinformatics* **17:** (7) 658.

Glusman G, Lancet D*. 2001. *Weizmann Inst Sci, Dept Mol Genet, IL-76100 Rehovot, Israel. Visualizing large-scale genomic sequences. *IEEE Eng Med Biol Mag* **20:** (4) 49.

Gorodkin J, Zwieb C, Knudsen B. 2001. Aarhus Univ, Inst Biol Sci, Dept Ecol & Genet, Bldg 540, DK-8000 Aarhus C, Denmark. Semi-automated update and cleanup of structural RNA alignment databases. *Bioinformatics* **17:** (7) 642.

Guha-Thakurta D, Stormo GD. 2001. Washington Univ, Sch Med, Dept Genet, 4566 Scott Ave, Campus Box 8232, St Louis, Mo 63110, USA. Identifying target sites for cooperatively binding factors. *Bioinformatics* **17:** (7) 608.

Kerr MK, Churchill GA*. 2001. *Jackson Lab, 600 Main St, Bar Harbor, Me 04609, USA. Bootstrapping cluster analysis: Assessing the reliability of conclusions from microarray experiments. *Proc Natl Acad Sci U S A* **98:** (16) 8961.

Lee JK. 2001. Univ Virginia, Sch Med, Dept Hlth Evaluat Sci, Div Biostat & Epidemiol, POB 800717, Charlottesville, Va 22908, USA. Analysis issues for gene expression array data. *Clin Chem* **47:** (8) 1350.

Lynn AM, Jain CK, Kosalai K, Barman P, Thakur N, Batra H, Bhattacharya A*. 2001. *Jawaharlal Nehru Univ, Bioinformatics Ctr, IN-110067 New Delhi, India. An automated annotation tool for genomic DNA sequences using GeneScan and BLAST. *J Genet* **80:** (1) 9.

Mills JC, Gordon JI*. 2001. *Washington Univ, Sch Med, Dept Mol Biol & Pharmacol, Box 8103, 660 Sth Euclid Ave, St Louis, Mo 63110, USA. A new approach for filtering noise from high-density oligonucleotide microarray datasets. *Nucleic Acids Res* **29:** (15) U5.

Mrowka R. 2001. Humboldt Univ, Charite, Johannes Muller Inst Physiol, Tucholsky Str 2, DE-10117 Berlin, Germany. A Java applet for visualizing protein protein interaction. *Bioinformatics* **17:** (7) 669.

Pasquier C, Promponas VJ, Hamodrakas SJ*. 2001. *Univ Athens, Fac Biol, Dept Cell Biol & Biophys, Panepistimiopolis, GR-15701 Athens, Greece. PRED-CLASS: Cascading neural networks for generalized protein classification and genome-wide applications. *Protein-Struct Funct Genet* **44:** (3) 361.

Sandberg R, Winberg G, Branden CI, Kaske A, Ernberg I, Coster J. 2001. Karolinska Inst, Ctr Microbiol & Tumor Biol, SE-17177 Stockholm, Sweden. Capturing whole-genome characteristics in short sequences using a naive Bayesian classifier. *Genome Res* **11:** (8) 1404.

Schaffer AA, Aravind L, Madden TL, Shavirin S, Spouge JL, Wolf YI, Koonin EV, Altschul SF. 2001. NIH, Natl Ctr Biotechnol Informat, 8600 Rockville Pike, Bethesda, Md 20894, USA. Improving the accuracy of PSI-BLAST protein database searches with composition-based statistics and other refinements. *Nucleic Acids Res* **29:** (14) 2994.

Steinfath M, Wruck W, Seidel H, Lehrach H, Radelof U, O'Brien J. 2001. Max-Planck-Inst Mol Genet Berlin Dahlem, Ihnestr 73, DE-14195 Berlin, Germany. Automated image analysis for array hybridization experiments. *Bioinformatics* **17:** (7) 634.

Wang WJ, Ghosh S, Guo SW. 2001. Med Coll Wisconsin, Max McGee Natl Res Ctr, Juvenile Diabet, 8701 Watertown Plank Rd, Milwaukee, Wi 53226, USA. Quantitative quality control in microarray image processing and data acquisition. *Nucleic Acids Res* **29:** (15) U32.

Copyright © 2002 John Wiley & Sons, Ltd. 
